# CD133+ cells derived from skeletal muscles of Duchenne muscular dystrophy patients have a compromised myogenic and muscle regenerative capability

**DOI:** 10.1016/j.scr.2018.05.004

**Published:** 2018-07

**Authors:** Jinhong Meng, Francesco Muntoni, Jennifer Morgan

**Affiliations:** a Dubowitz Neuromuscular Centre, Molecular Neurosciences Section, Developmental Neuroscience Programme, UCL Great Ormond Street Institute of Child Health, 30 Guilford Street, London WC1N 1EH, UK; b NIHR Great Ormond Street Hospital Biomedical Research Centre, 30 Guilford Street, London WC1N 1EH, UK

**Keywords:** CD133+ cells, Muscle stem cells, DMD, Muscle regeneration

## Abstract

Cell-mediated gene therapy is a possible means to treat muscular dystrophies like Duchenne muscular dystrophy. Autologous patient stem cells can be genetically-corrected and transplanted back into the patient, without causing immunorejection problems. Regenerated muscle fibres derived from these cells will express the missing dystrophin protein, thus improving muscle function.

CD133+ cells derived from normal human skeletal muscle contribute to regenerated muscle fibres and form muscle stem cells after their intra-muscular transplantation into an immunodeficient mouse model. But it is not known whether CD133+ cells derived from DMD patient muscles have compromised muscle regenerative function.

To test this, we compared CD133+ cells derived from DMD and normal human muscles. DMD CD133+ cells had a reduced capacity to undergo myogenic differentiation *in vitro* compared with CD133+ cells derived from normal muscle.

In contrast to CD133+ cells derived from normal human muscle, those derived from DMD muscle formed no satellite cells and gave rise to significantly fewer muscle fibres of donor origin, after their intra-muscular transplantation into an immunodeficient, non-dystrophic, mouse muscle.

DMD CD133+ cells gave rise to more clones of smaller size and more clones that were less myogenic than did CD133+ cells derived from normal muscle. The heterogeneity of the progeny of CD133+ cells, combined with the reduced proliferation and myogenicity of DMD compared to normal CD133+ cells, may explain the reduced regenerative capacity of DMD CD133+ cells.

## Highlights

• The myogenicity of CD133+ cells from Duchenne muscular dystrophy skeletal muscle is compromised.
• Duchenne muscular dystrophy CD133+ cells regenerate skeletal muscle less than normal CD133+ cells.
• Skeletal muscle-derived CD133+ cells consist of myoblasts, pericytes and fibroblasts.

## Graphical abstract

**Figure:**
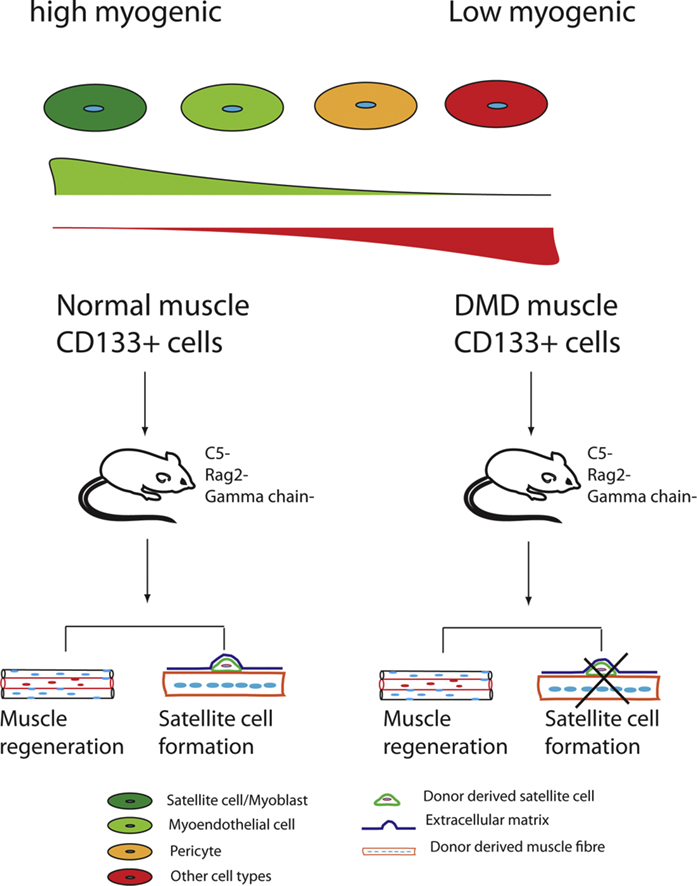

## 1 Introduction

CD133+ cells are a rare population that resides within human skeletal muscle (Benchaouir et al., 2007; Meng et al., 2014; Negroni et al., 2009). We and others have shown that CD133+ cells isolated from normal human muscle contribute to muscle regeneration (Negroni et al., 2009) and form functional muscle stem cells after their intra-muscular transplantation in an immunodeficient mouse model (Meng et al., 2014), but CD133+ cells derived from DMD patient muscle have not been extensively investigated (Benchaouir et al., 2007; Meng et al., 2014).

The use of autologous, rather than heterologous, donor stem cells will reduce the possibility of their immunological rejection, although they will have to be genetically modified prior to their transplantation (Counsell et al., 2017; Meng et al., 2016; Young et al., 2016). But satellite cells from DMD muscle may not function correctly, due either to lack of dystrophin expression at the required time (Dumont et al., 2015), or the fact that continual activation of stem cells in pathological muscle may lead to their exhaustion (Blau et al., 1983; Sacco et al., 2010; Mouly et al., 2005). In addition, changes in the components of the stem cell niche (Smith et al., 2016; Alexakis et al., 2007; Sabatelli et al., 2012) (*e.g.* alterations in components of connective tissue, or of the muscle fibre) or signalling pathways (Jiang et al., 2014) may be deleterious to satellite cell function. It is not known whether any of these factors affect CD133+ cells.

We therefore decided to compare the myogenicity and muscle regenerative capacity of CD133+ cells derived from the muscles of 4 control and 4 DMD patients with different mutations in the *DMD* gene. DMD CD133+ cells had impaired myogenic capacity both *in vitro* and *in vivo*. Clonal analysis suggested that this may relate to the increased proportion of slow proliferating colonies and low/non-myogenic colonies within the CD133+ cell -derived clones from DMD compared to normal muscles. We also found that human skeletal muscle -derived CD133+ cells are heterogeneous, containing cells of satellite cell/myoblast, myoendothelial, pericyte and fibroblast lineages.

Although CD133+ isolated from normal human muscle contribute to muscle regeneration *in vivo*, their counterpart from DMD patients are significantly less effective. This will have to be taken into consideration when thinking of using stem cells derived from patient muscle therapeutically.

## 2 Results

### 2.1 CD133+ cells derived from DMD muscles have variable myogenicity *in vitro*

**Fig. 1:**
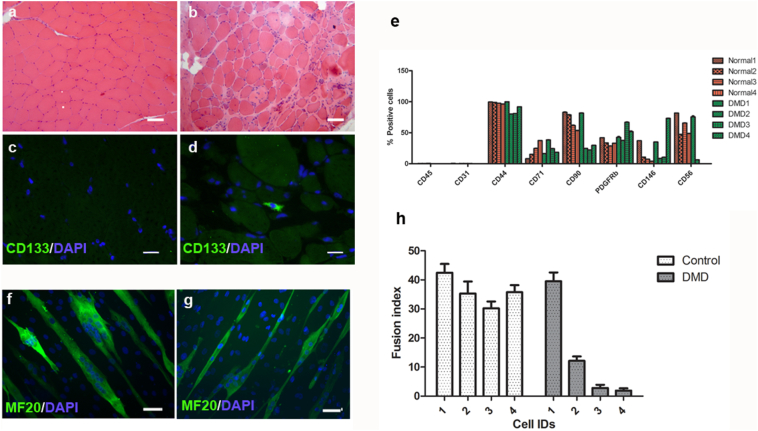
Location of CD133+ cells within human skeletal muscle, characterization of CD133+ cell population and their myogenic capacity *in vitro*. a,b: H&E staining of normal and DMD human skeletal muscle sections show typical degeneration and regeneration muscle fibres in DMD muscle biopsy (b), in comparison to normal muscle biopsy (a). Scale bar = 25 μm. c, d: Immunofluoresent staining of CD133 on normal (c) and DMD (d) muscle sections. There were CD133+ cells present within DMD muscles. Scale bar = 10 μm. e: Characterization of CD133+ cell population by FACS analysis of cell surface markers. Skeletal muscle-derived CD133+ cells lack haematopietic stem cell and endothelial cell markers. All cell populations contained cells expressing mesenchymal stem cell and pericyte markers. Not all DMD cell populations contain CD56+ cells. f, g: *In vitro* myogenicity of CD133+ cells. Four normal and four DMD CD133+ cell preparations were induced to undergo myogenic differentiation *in vitro*. Myotubes were stained with an antibody to myosin heavy chain (MF20, green), nuclei were counterstained with DAPI (blue). f and g are representative images of the MF20 staining of DMD1 and DMD2 cells. Scale bar = 25 μm. h: fusion index (mean ± SEM) of each cell preparation. (For interpretation of the references to colour in this figure legend, the reader is referred to the web version of this article.)

We have shown that CD133+ cells isolated from normal human muscle are myogenic *in vitro* and can contribute to muscle regeneration in an *in vivo* mouse model (Meng et al., 2014; Meng et al., 2015). In order to investigate CD133+ cells from DMD muscle, we performed H&E and immunostaining of CD133 on skeletal muscle sections from either normal (n = 2) or DMD patients (n = 3). The details of muscle biopsies used in this experiment are listed in Table 1. As expected, normal muscles stained with H&E had little fat or fibrotic tissue, while DMD muscles had pathological changes typical of DMD (Fig. 1a, b). In line with our previous finding (Meng 2014), CD133+ cells were in the satellite cell position in muscle biopsies from 18-day old infants (Meng et al., 2014), but not in normal biopsies from individuals older than 2-years of age (Fig. 1c). However, in 2 out of 3 muscle biopsies from DMD patients, CD133+ cells were found outside the myofibres (Fig. 1d and Table 1). These data suggest that the composition of CD133+ cells in normal and DMD muscles may not be the same, thus there might be functional differences between normal and DMD CD133+ cells.

**Table 1:** Muscle biopsies used for H&E and immunostaining.

Donor	Muscle	Age (years)	Mutation	H&E	IF of CD133
Normal	Quadriceps	3	None	Morphologically normal	ND
Normal	Quadriceps	9	None	Morphologically normal	ND
DMD	Quadriceps	5	Dystrophin point mutation, c.3804G > A (p.Trp 1268X), detected in exon 28	Features of DMD pathology	ND
DMD	Quadriceps	8	Dystrophin point mutation, c.9851G > A (p.Trp 3284X), detected in exon 68	Features of DMD pathology	Present
DMD	Quadriceps	4	Dystrophin point mutation, c.1388G > A (p.Trp 463X), detected in exon 12	Features of DMD pathology	Present

*ND: Not detected.

To investigate the myogenic capacity of CD133+ cells derived from the muscles of DMD patients, we isolated these cells from 4 DMD patients and 4 normal donors (Table 2) and expanded them in M10 medium. We performed FACS analysis of all the normal and DMD CD133+ preparations at mpds from 14.38–18.47 (Fig. 1e). As expected, we found that all were negative for CD45 and CD31, suggesting there were no cells of blood or endothelial origin. All cell preparations contained cells expressing mesenchymal stem cell lineage markers (CD44, CD71) and pericyte markers (CD146, PDGFRβ). However, we found that CD56, a myogenic cell marker, was differentially expressed. In highly myogenic cell preparations (*i.e.* normal CD133+ cells and DMD1 CD133+ cells), the percentage of CD56+ cells was above 50%; DMD2, which was less myogenic, had 6.32 ± 0.38% CD56+ cells. The non-myogenic cell preparations DMD3 and DMD4 contained no CD56+ cells. Overall, our data suggest that all the CD133+ cell preparations contain cells that express typical mesenchymal stem cell surface markers. The extent of CD56 expression seems to correlate with the myogenicity of the cell preparation.

**Table 2:** Cell preparations used in this study.

Donors	Age (years)	Muscle	Mutation	*In vitro* myogenesis (Fusion index)	Clonal culture	*In vivo* transplantation
DMD1	18	Paraspinal	Dup Ex56	39.53 ± 3.04%	Yes	Yes
DMD2	11	Quadriceps	Frameshift mutation in Exon34, c4686-4689del (p.Arg 1562 fs)	12.13 ± 1.49%	Yes	Yes
DMD3	9	Quadriceps	Del 49–50	1.89 ± 0.79%	Yes	ND
DMD4	13	Quadriceps	Point mutation in Exon67	2.82 ± 1.06%	ND	ND
Normal 1	2	Paraspinal		42.46 ± 3.0%	ND	Yes
Normal 2	14	Paraspinal		35.30 ± 4.15%	ND	ND
Normal 3	15	Paraspinal		30.23 ± 2.32%	ND	ND
Normal 4	15	Quadriceps		35.79 ± 2.42%	ND	ND
Normal 5	5	Paraspinal		ND	Yes	ND

ND: not determined.

Next, the myogenic capacity of the cell populations was evaluated *in vitro* by inducing them to undergo myogenic differentiation (Meng et al., 2011; Meng et al., 2014). We found that not all of the DMD CD133+ cell preparations were myogenic *in vitro*. Of the four DMD cell preparations investigated, two were virtually non-myogenic, with a fusion index (FI) of <3%, and the other two preparations had fusion indexes of 39.53 ± 3.04% and 12.13 ± 2.97%, respectively (Fig. 1 f, g). In contrast, all four preparations of CD133+ cells derived from normal muscle were myogenic *in vitro*, with fusion indexes ranging from 30.23 ± 2.32% to 42.5 ± 3.0% (Fig. 1h). Overall, DMD CD133+ cell preparations are more variable in terms of their *in vitro* myogenic differentiation than normal CD133+ cells.

### 2.2 Some DMD CD133+ cell preparations contribute to regenerated muscle fibres, but do not form satellite cells, *in vivo*

**Fig. 2:**
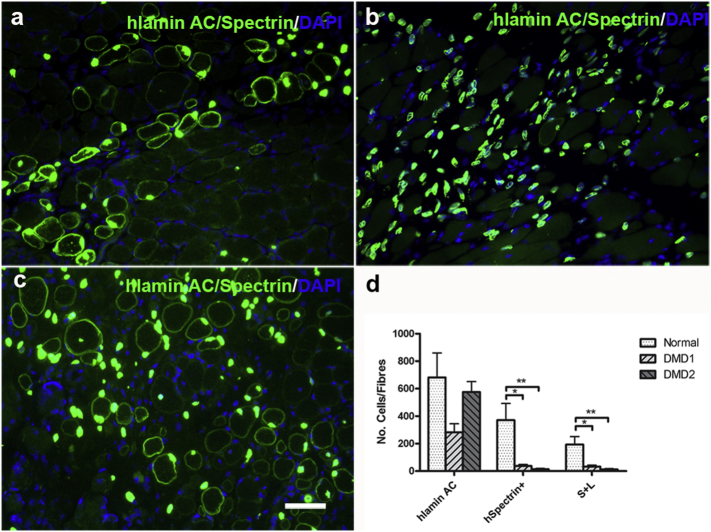
Contribution of DMD and normal CD133+ cells to muscle regeneration. a–c are representative images showing the nuclei (human Lamin A/C+) and muscle fibres (human spectrin+) of donor origin in representative transverse cryosections of muscles that had been transplanted with DMD1 (a), DMD2 (b) and normal CD133+ cells (c). Scale bar = 25 μm. d shows the quantification and comparison of the number (mean ± SEM) of cells/fibres of donor origin in each transplantation group. *p < 0.05; **p < 0.01.

We tested the contribution of one normal and the two DMD CD133+ cell preparations that were myogenic *in vitro* to muscle regeneration and satellite cell formation in an *in vivo* mouse model. One DMD CD133+ cell preparation (DMD1) formed regenerated muscle fibres (human Spectrin+ fibres: 37.33 ± 10.6; fibres expressing human spectrin and containing at least one human lamin a/c + nucleus (S + L fibres): 33.3 ± 9.6 Mean ± SEM, n = 6) after intra-muscular transplantation (Brimah et al., 2004; Meng et al., 2014; Meng et al., 2015; Silva-Barbosa et al., 2005; Silva-Barbosa et al., 2008) into Rag2-/γ chain-/C5- immunodeficient mice. Although DMD2 was myogenic *in vitro* (FI = 12.13 ± 2.97%) and gave rise to cells of donor origin within the host muscles (575.4 ± 75.5 human lamin AC+ nuclei, Mean ± SEM, n = 7), they contributed to very little muscle regeneration after transplantation (human spectrin + fibres: 13.86 ± 5.7 and S + L fibres 12.4 ± 5.5, Mean ± SEM, n = 7). Consistent with our previous findings (Meng et al., 2014; Meng et al., 2015), the normal CD133+ cell preparation contributed to regenerated muscle fibres (human spectrin+ fibres: 371.7 ± 120.8, S + L fibres:193.5 ± 57.98, Mean ± SEM, n = 6) after transplantation (Fig. 2). The two DMD CD133+ cell preparations therefore contributed to significantly less muscle regeneration than the CD133+ cells derived from normal muscle.

**Fig. 3:**
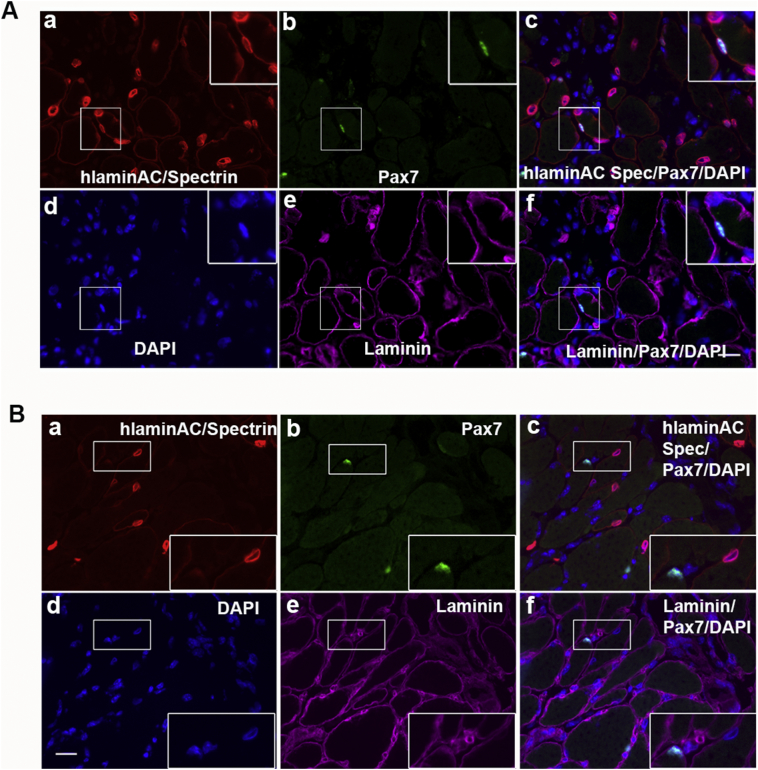
Contribution of CD133+ cells to the satellite cell pool after intramuscular transplantation. A: Normal CD133+ cells contribute to satellite cell pool after transplantation. Representative images showing satellite cells (Pax7+, green), which were located underneath the basal lamina (Laminin, magenta) and of donor normal CD133+ origin (human lamin AC+ nuclei, red). B. Representative images showing that donor DMD CD133+ cells did not give rise to satellite cells. Note that there are Pax7+ cells (green) of mouse host origin (negative for human lamin AC) present in the transplanted muscle. None of the donor nuclei (human lamin AC+) colocalized with Pax7, evidence that DMD CD133+ cells did not contribute to satellite cell formation after transplantation. Transverse section stained with antibodies against Pax7 (green), human lamin AC and human spectrin (both red) and laminin (magenta). Nuclei were counterstained with DAPI (blue). Scale bar = 10 μm. (For interpretation of the references to colour in this figure legend, the reader is referred to the web version of this article.)

Next, we tested whether the DMD CD133+ cells with the greatest muscle regenerative capacity (DMD1) formed satellite cells after transplantation. In line with our previous findings (Meng et al., 2014, Meng et al., 2015), after transplantation of normal CD133+ cells, there were human lamin AC+/Pax7+ cells underneath the basal lamina of the muscle fibres (Fig. 3A); on the contrary, there were no human lamin AC+/Pax7+ cells (satellite cells or myoblasts of donor origin) in muscles that had been transplanted with the DMD1 CD133+ cells (Fig. 3B), implying that DMD CD133+ cells were unable to form satellite cells after intramuscular transplantation.

Our *in vitro* and *in vivo* assays suggest that CD133+ cells derived from DMD muscle have a compromised capacity to contribute to muscle regeneration and that this may be related to their low myogenic capacity *in vitro*.

### 2.3 CD133+ cells from DMD muscle gave rise to a higher percentage of slow-growing or non-proliferative colonies than CD133+ cells from normal muscle

**Supplementary Fig. 1:**
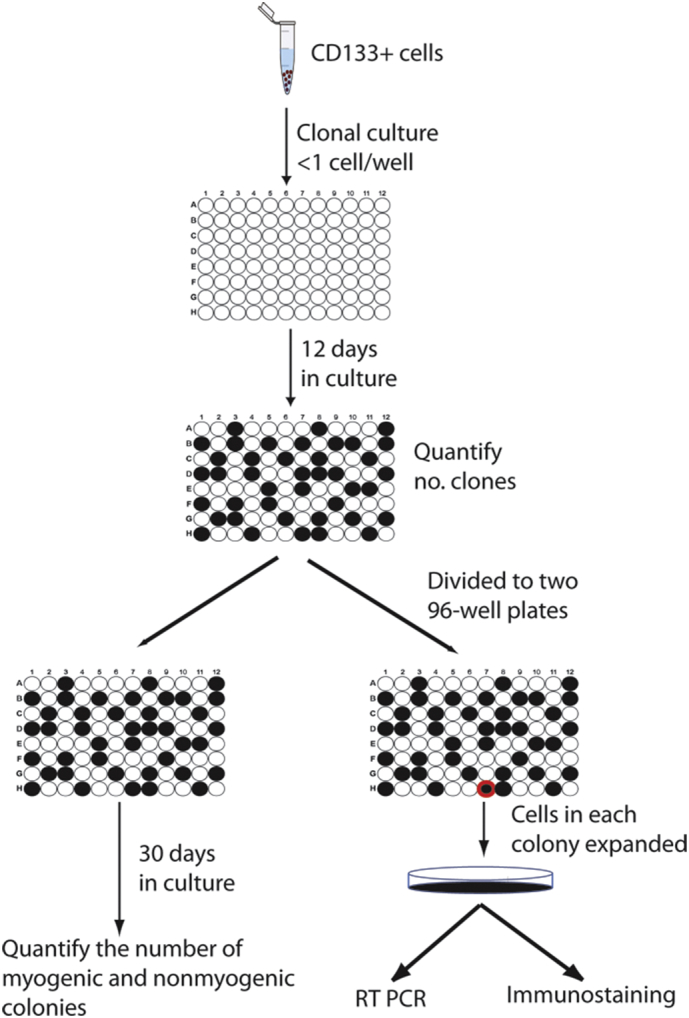
Schematic illustration of the experimental procedure for the clonal culture of CD133+ cells. Freshly isolated human skeletal muscle CD133+ cells were plated at clonal density in 96-well plates. Cell clones were visualized at 12 days after culture. The number of total, small, medium and large clones were counted and the cells in 96-well plate were trypinized and divided into duplicate wells of two new 96 well plates. One 96 well plate was maintained for 30 days and the number of myogenic and nonmyogenic clones were counted. Individual clones in the replicate 96 well plate were trypsinized to larger vessels once they become sub-confluent, and cells expanded. RNA was then extracted from the clones for RT-PCR analysis. Cells were also plated onto poly-l-lysine coated coverslips for immunofluorensent analysis.

To investigate the possible reason for the compromised myogenic and regenerative capacity of the DMD CD133+ cells, we performed clonal analyses (Supplementary Fig. 1) of the freshly isolated CD133+ cells from normal and DMD skeletal muscles.

**Fig. 4:**
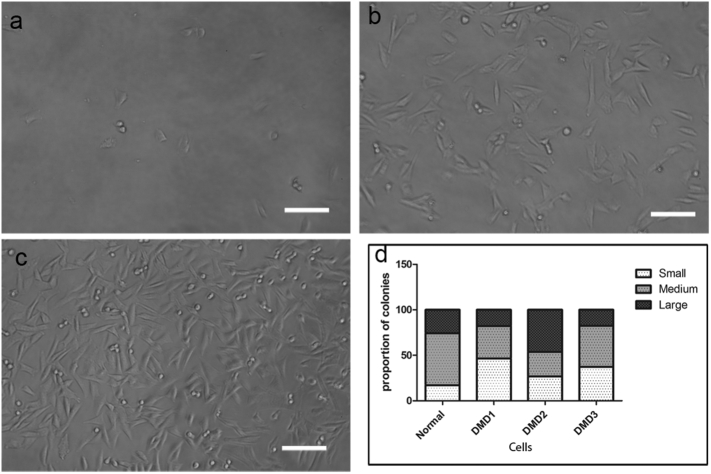
CD133+ cell populations give rise to different sized colonies. a–c: representative images of small (a), medium (b) and large (c) colonies in culture. Scale bar = 100 μm. D: the proportion of different sized colonies derived from normal and DMD CD133+ cells.

After 12 days in culture, colonies consisted of different numbers of cells (Fig. 4), suggesting that they proliferated at different rates. Medium colonies contained 200 to 5 × 10^4^ cells (Fig. 4b). Large colonies contained >5 × 10^4^ cells, most of them having reached confluence (Fig. 4c), suggesting they were proliferating at a higher rate than other colonies. Small colonies contained <200 cells after one month in culture (Fig. 4a); as cells in these colonies could not be expanded further, no RT-PCR or immunostaining analysis were performed on this type of clones.

We quantified the number of small, medium and large colonies in 3 DMD CD133+ cell preparations and one normal CD133+ cell preparation. As shown in Fig. 4d, all the DMD CD133+ cell preparations contained a higher percentage (36.87 ± 5.6%, n = 3) of small colonies than did the normal CD133+ preparation (17.14%), suggesting that more of the DMD CD133+ cells had a limited proliferation capacity.

### 2.4 DMD CD133+ cells gave rise to more colonies with low myogenicity than normal CD133+ cells

**Fig. 5:**
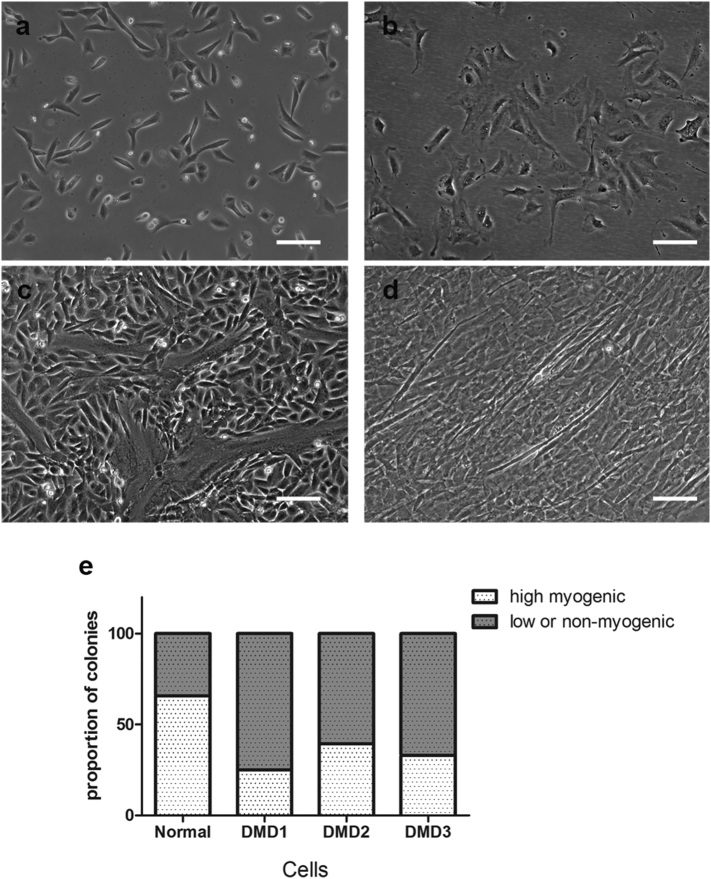
Colonies with different myogenic capacities are present within the CD133+ cell population. a showed the typical morphology of the high myogenic colonies in culture. These cells underwent spontaneous differentiation to form large myotubes (c). b shows the typical morphology of the low/non-myogenic colonies in culture. These cells had limited myogenic capacity: some could form a few, sparse myotubes (d), whereas others did not form myotubes in culture (not shown). Scale bar = 100 μm. e quantification of the proportion of the high and low/non-myogenic colonies within the normal and DMD cells preparations.

In addition to the proliferation rate, each preparation contained colonies of different morphologies. Some high myogenic colonies contained cells which are small, refractile, with no or very short processes (Fig. 5a). All of these colonies underwent spontaneous myogenesis during the one month cultivation period, with myotubes present in proliferation medium (Fig. 5c). The morphology of cells in other colonies was noticeably different: cells were flat and less refractile (Fig. 5b). Some of these colonies underwent spontaneous myotube formation during the one month cultivation period, but to a much lesser extent than the high myogenic colonies (Fig. 5d), thus was classified as low-myogenic colonies. Some colonies did not generate myotubes in culture (not shown) and were therefore classified as non-myogenic.

We quantified the number of high and low/non-myogenic colonies in each cell preparation. As shown in Fig. 5e, there was a higher proportion of low or non-myogenic colonies in the 3 DMD cell preparations (67.3 ± 6.1%), in comparison to the normal CD133+ cell preparation (34%).

### 2.5 Molecular signature of the different types of colony derived from normal CD133+ cells

To determine the molecular signature of the different types of colonies, we extracted mRNA from randomly-selected colonies that were high, low, or non-myogenic at the time when they were in their expansion phase. We did not include in these analyses colonies that did not express CD133 at the RNA level.

**Fig. 6:**
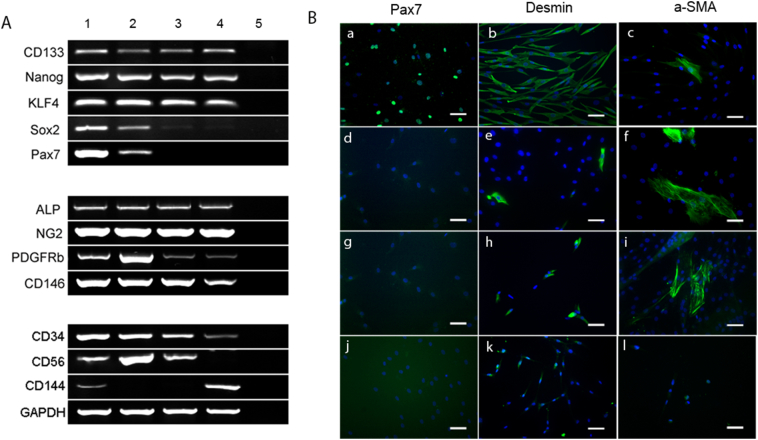
Characterization of different types of colonies within the normal CD133+ cell population. A: semi-quantitative RT-PCR showing the expression of the cell lineage markers in a representative high myogenic colony (lane 1), low myogenic colonies (lane2 and 3) and non-myogenic colony (lane4). Lane5 is the water only control for RT-PCR. B: Immunostaining of Pax7, Desmin, and α-SMA on high colony (a–c), low (d–g) and non-myogenic colonies (j–i). Nuclei were counter stained with DAPI (blue). Scale bar = 25 μm. (For interpretation of the references to colour in this figure legend, the reader is referred to the web version of this article.)

We performed semi-quantitative RT-PCR on mRNA from the CD133+ colonies with early stem cell markers nanog, KLF4 and Sox2. All CD133+ colonies expressed similar amounts of early stem cell markers nanog and KLF4, suggesting that CD133+ cell are primitive stem cells within human skeletal muscle. Sox2 expression differed amongst the colonies: it was highest in high myogenic colonies (Fig. 6a, lane 1), slightly lower in the low myogenic colonies (Fig. 6a, lane 2 and 3) and lowest in non-myogenic colonies (Fig. 6a, lane 4). Interestingly, Sox2 expression correlated with Pax7 expression. We found that all the high myogenic colonies expressed high levels of the satellite cell marker Pax7 (Fig. 6a, lane 1); low myogenic colonies were either Pax7+ (Fig. 6a, lane 2) or Pax7- (Fig. 6a, lane 3) and, as might be expected, non-myogenic colonies did not express Pax7 at all (Fig. 6a, lane 4). The fact that some of the colonies express Pax7 suggests that some CD133+ cells are satellite cells, which correlates with our immunostaining results on human muscle sections (Meng et al., 2014).

Nearly all colonies expressed the pericyte markers ALP, NG2, PDGFRβ and CD146 (Crisan et al., 2008; Dellavalle et al., 2007). There were no differences in the expression levels of NG2 and ALP, whilst expression of PDGFRβ and CD146 showed some variation amongst colonies. The presence of pericyte markers suggested that either most, if not all, CD133+ cells were pericytes, or that they or their progeny were able to differentiate along the pericyte lineage.

All myogenic colonies expressed CD34 and CD56. CD144, an endothelial marker, was only expressed by high myogenic (Fig. 6A lane 1) and non-myogenic (Fig. 6A, lane 4) colonies. These data suggest that some of the high myogenic colonies may either be derived from myoendothelial cells (Zheng et al., 2007; Okada et al., 2008), or able to differentiate into the myoendothelial cell lineage.

### 2.6 Phenotype of the different types of colony derived from CD133+ cells

**Supplementary Fig. 2:**
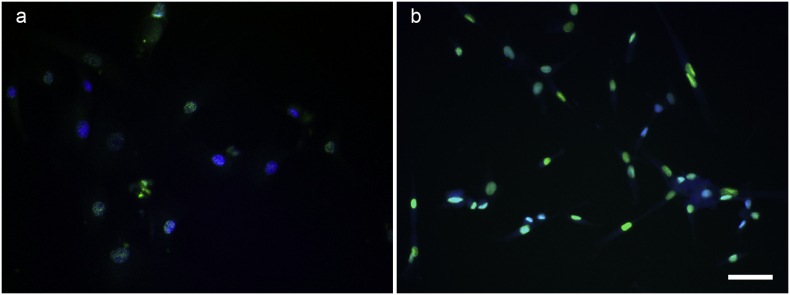
immunostaining of TCF4 (green) on high (a) and non- myogenic (b) clones. Nuclei were counterstained with DAPI (blue). Scale bar = 25 μm.

Next, we performed immunostaining on high, low and non-myogenic colonies derived from normal CD133+ cells, to compare their phenotypes. High myogenic colonies contained high percentages of Pax7+ and desmin+ cells. In addition, cells expressing pericyte markers such as ALP, PDGF-β receptor, and α-SMA were also present in high myogenic colonies. In low myogenic colonies, no Pax7+ cells were present, even in colonies which expressed Pax7 at the mRNA level. These colonies contained fewer desmin+ cells than the high myogenic colonies. Low myogenic colonies also contained cells expressing pericyte markers ALP, PDGF-β receptor and α-SMA. In non-myogenic colonies, none of the above myoblast or pericyte markers was present at the protein level. However, when stained with a fibroblast cell marker, TCF4 (Supplementary Fig. 2), the non-myogenic clone had strong nuclear staining of TCF4 (Mathew et al., 2011), suggesting these cells might be fibroblasts.

**Fig. 7:**
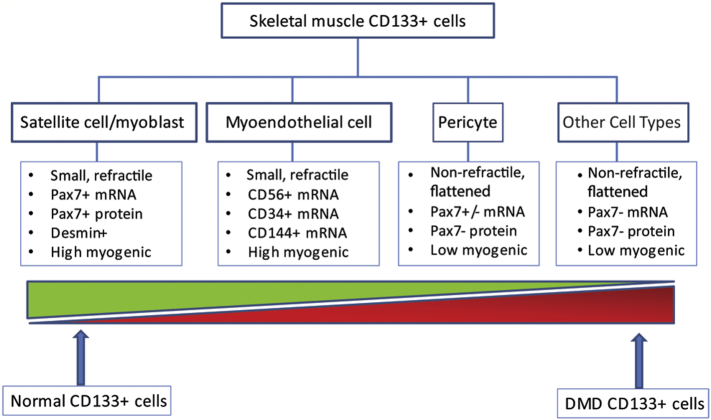
Schematic illustration of the heterogeneity of the skeletal muscle CD133+ cells and the differences in the proportion of high or low/non-myogenic colonies in normal and DMD muscles. CD133+ cells derived from human skeletal muscle contained cells of satellite cell/myoblast, myoendothelial, pericyte and other cell lineages, with different morphologies and myogenic capacities. The proportion of the high myogenic cells is greater in clones derived from normal CD133+ cells in comparison to DMD CD133+ cells.

The molecular signature and the phenotype of the colonies suggested that human CD133+ cells either are, or give rise to, a heterogonous cell population containing both myogenic and non-myogenic cells and that the highly myogenic cells are closely related to the satellite cell/myoblast lineage, low myogenic cells more related to the pericyte lineage, while the non-myogenic cells tend to be other interstitial cells including fibroblast-like cells (Fig. 7).

## 3 Discussion

Successful stem cell therapy relies on highly potent cells of the correct phenotype. There are many types of muscle stem cell capable of contributing to muscle regeneration (Benchaouir et al., 2007; Brimah et al., 2004; Dellavalle et al., 2007; Zheng et al., 2007). Amongst these, skeletal muscle-derived myoblasts and CD133+ cells can reconstitute the muscle stem (satellite cell) pool, giving the possibility of longer term treatment to the patients (Meng et al., 2014; Skuk et al., 2010). Donor stem cells may either be heterologous, or genetically-modified to produce the missing protein.

Autologous stem cell transplantation is superior to allografts, due to lack of immune-rejection issues. But DMD myoblasts have defects in their proliferation capacity, limiting their applicability for cell-mediated therapy (Blau et al., 1983). It is not clear if CD133+ cells derived from skeletal muscles of DMD patients have equivalent muscle regenerative ability to CD133+ cells derived from non-dystrophic donors. Using immunostaining, we found no CD133+ cells in normal muscle sections (n = 2), although we managed to isolate CD133+ cells from biopsies of normal muscle. However, this is not unexpected, as CD133+ cells are so rare within skeletal muscle, which might explain why none were found in a single 7 μm transverse section of the biopsy. In keeping with this, in our previous study (Meng et al., 2014), we also found no CD133+ cells in muscle sections from normal donors over 2 years of age. We only found CD133+ cells in muscle sections from very young donors (18 days of age, n = 2). Nevertheless, we isolated CD133+ cells from muscle biopsies of 4 DMD patients with different mutations; CD133+ cells isolated from normal donors were used as controls. FACS analysis confirmed that none of these preparations contained cells of haematopoetic or endothelial origin and all contained cells expressing mesenchymal stem cell and pericyte markers. Interestingly, all the normal CD133+ cell preparations contained cells expressing the marker CD56 (Uezumi et al., 2016), while only 2 DMD cell preparations (DMD1 and DMD2) contained any CD56+ cells (Fig. 1g). The percentage of CD56+ cells appears to correlate with the fusion index of the cell preparation. Further evaluation of the myogenic capacity of these 4 DMD cell populations showed that, in contrast to CD133+ cells derived from skeletal muscle of normal donors, which were all highly myogenic *in vitro* (Fig. 2), there were large variations in myogenicity amongst the DMD cell preparations. The DMD populations (DMD1 and DMD2) that contained higher percentages of CD56+ cells were more myogenic than populations (DMD3 and DMD4) that contained either far less, or no, CD56+ cells.

Our findings illustrate that, similarly to myoblasts, CD133+ cells derived from DMD skeletal muscle may have a reduced myogenic capacity. Although previous studies have shown that the deficiencies in DMD myoblasts were not due to the primary genetic defect (Webster et al., 1986), recent work has shown that dystrophin expression on activated satellite cells in mouse muscle affected their asymmetric division (Dumont et al., 2015), suggesting that lack of dystrophin adversely affects muscle stem cell function. Our previous finding (Meng et al., 2014) in 18-day old normal muscle biopsies showed that some CD133+ cells are present in the satellite cell position (underneath the basal lamina of muscle fibres), suggesting that there is an overlap between CD133+ cells and satellite cells. Interestingly, in DMD muscles, most CD133+ cells are in the interstitial space between muscle fibres (therefore not satellite cells). The fact that CD133+ cells in DMD muscles are cells other than satellite cells (Fig. 1) (Meng et al., 2014), is a possible explanation of the attenuated myogenic and regenerative capacity of CD133+ cells derived from skeletal muscle of DMD patients.

Indeed, clonal analysis of freshly isolated CD133+ cells from DMD and normal muscle showed that DMD CD133+ cells gave rise to a higher percentage of small and low/non-myogenic colonies than normal CD133+ cells. This may result from the chronic pathological changes within DMD muscle. As low or non- myogenic colonies contained more cells than the high myogenic colonies, a bulk CD133+ preparation derived from DMD muscle may be overwhelmed by non-myogenic cells after *in vitro* expansion.

In addition, normal human skeletal muscle-derived CD133+ cells are heterogeneous, containing progenitors of myoblasts, myoendothelial cells, pericytes and fibroblasts. Cells that gave rise to high myogenic colonies may be multipotent stem cells, as clones derived from these cells express markers of all these cell lineages at the mRNA level, and myoblast (Pax 7 and desmin) and pericyte (α-SMA) markers at the protein level. Low myogenic colonies may originate from stem cells with the potential to differentiate along the pericyte lineage. But some of these low myogenic colonies expressed Sox2 and Pax7 at the mRNA level (Fig. 6A, lane 2) and contained desmin+ cells (Fig. 6B, d–f), suggesting that they have some myogenic potential. Non-myogenic colonies expressed the lowest levels of myoblast and pericyte markers but expressed the endothelial marker CD144 at the mRNA level, suggesting that cells that give rise to these types of colony could be the progenitor of fibroblasts or endothelial cells within human muscle.

## 4 Conclusions

CD133+ cells isolated from DMD patients are not as myogenic as their counterpart derived from normal skeletal muscle. One possible reason for this might be that chronic pathological changes within DMD muscle caused an upregulation of CD133 expression on non-myogenic cells within the muscle, thus increasing the proportion of the non-myogenic cells within the progeny of CD133+ cells. This must be taken into account if considering the use of CD133+ cells derived from DMD patient muscle as an autologous stem cell for future clinical application.

## 5 Material and methods

### 5.1 Ethics

Tissue sampling was approved by the NHS national Research Ethics Service, Hammersmith and Queen Charlotte's and Chelsea Research Ethics Committee: Setting up of a rare diseases biological samples bank (biobank) for research to facilitate pharmacological, gene and cell therapy trials in neuromuscular disorders (NMD) REC reference number: 06/Q0406/33 and the use of cells as a model system to study pathogenesis and therapeutic strategies for Neuromuscular Disorders (REC reference 13/LO/1826), in compliance with national guidelines regarding the use of biopsy tissue for research. All patients or their legal guardians gave written informed consent.

Animal experiments have completed UCL's ethical review process. Mice were bred and experimental procedures were carried out in the Biological Services Unit, University College London Great Ormond Street Institute of Child Health, in accordance with the Animals (Scientific Procedures) Act 1986. Experiments were performed under Home Office licence numbers 70/7086 and 8389. Experiments were approved by the local University College London ethical committee prior to the licence being granted.

#### 5.1.1 Hematoxylin and eosin (H&E) staining and immunostaining of CD133 on muscle sections

Biopsies of quadriceps muscles of 3 DMD patients and 2 normal controls were used (Table 1). Serial 7 μm cryosections were stained with H&E and an antibody against human CD133 (Milteni biotech, clone 293C3). For H&E staining, the sections were air dried before being stained with hematoxylin and eosin. The sections were dehydrated through 30%, 50%, 75%, 95% and 100% ethanol, followed by xylene for 10 min. The sections were mounted using DPX mounting solution. For immunostaining of CD133, muscle sections were air dried before being incubated in primary antibody (CD133, 1:100, diluted in PBS containing 10% normal goat serum and 0.03% Triton ×100) for 2 h at room temperature (RT). After rinsing with PBS 3 × 5 min, the sections were incubated with Alexa-488 conjugated goat anti-mouse IgG (H + L) antibody (1,1000, Thermo Fisher) for 1 h at RT. The sections were rinsed with PBS for 5 min and then mounted with DAKO mounting medium (containing 10 μg/ml DAPI).

#### 5.1.2 Isolation and characterization of CD133+ cells

Biopsies of para-spinal or quadriceps muscle of 4 DMD patients and 4 normal control were taken with the patient's consent (Table 2). Muscles were digested with an enzyme mixture containing collagenase IA-S (Sigma, C9722, Dorset, UK), II (Sigma, C1764, Dorset, UK) and IV (Sigma, C1889, Dorset, UK) (1 mg/ml each) in 20% fetal bovine serum (FBS) DMEM for 45 min at 37°C. The cell suspension was then filtered through a 40 μm cell strainer (SLS, 352340, Nottingham, UK) and centrifuged at 300*g* for 10 min at RT. The cells were re-suspended in PBS and incubated with anti-human CD133 microbeads (Miltenyi Biotec, 130-050-801 Surrey, UK), 1:11 at 4°C for 30 min. The CD133+ and CD133- cell populations were separated using LS column (Miltenyi Biotec, 130-042-401, Surrey, UK) in a MACS system (Miltenyi Biotec, Surrey, UK). Resulting CD133+ cells were cultured in M10 medium containing Megacell DMEM (M3942, Sigma, Dorset, UK), 10% fetal bovine serum (FBS), 2μM glutamine, 1% non-essential amino acids (NEAA), 0.1 mM β-mercaptoethanol (β-ME) and 5 ng/ml basic fibroblast growth factor (bFGF) on 0.2% gelatin coated 6 well plate, at density of 100–500 cells per well. CD133+ cells usually approached confluence 5 days after initiating the culture. For maintenance, cells were passaged and plated onto 1 mg/ml collagen I coated 75 cm^2^ flasks at a density of 2.5 × 10^5^ cells/flask. Cells were passaged every 3–4 days.

For characterization of CD133+ cell populations, cultured cells were trypsinized and centrifuged at 500*g* for 5 min at room temperature. 5 × 10^5^ cells were used for each FACS analysis. Cells were rinsed with PBS and blocked for 30′ with 10% bovine serum albumin (BSA) in PBS, before being incubated with PE-conjugated primary antibodies (CD44:PE, CD45:PE, CD90:PE, CD56:PE) for one hour. PE conjugated isotype -matched negative antibodies (IgG1: PE or IgG 2a: PE, 1:20, Bio-Rad, UK) were used as controls. For CD71, CD31, PDGFRβ (1,50, Bio-Rad, UK) staining, cells were incubated with primary antibodies for 1 h, followed by PE-conjugated rabbit anti mouse IgG (H + L) (1,20, Bio-Rad, UK) secondary antibody. Cells incubated with PE-conjugated rabbit anti mouse IgG (H + L) antibody served as control. A BD LSRII FACS machine was used for acquiring the data, and 10, 000 events were collected each sample. Flowjo 7.2.5 software was used to analyse the results.

#### 5.1.3 Clonal culture of hCD133+ cells

Freshly isolated CD133+ cells were plated onto 0.2% gelatin coated 96-well plates at a density of <1 cell/well, and cultured in M10 medium. Colonies became visible at around 10–12 days after culture. The plate was then trypsinized and the cells from each well divided into two 96-well plates, using a multichannel pipette. One plate was reserved for observing the spontaneous myogenic potential of each colony after long-term (one month) *in vitro*, medium was changed every 3–4 days; the other plate was manipulated as follows: 1). Clonal formation and relative proliferation rate was analysed 12 days after plating. A well containing cells was considered as a colony. According to their size, colonies were defined as being small (<200 cells), medium (from 200 cells to 60% confluence) and large (over 60% confluence) colonies. 2). Colonies were dissociated with Trypsin:EDTA when they reached 50% confluency and expanded into 24-well plates to generate sufficient cells for RT-PCR assay and immunostaining analysis. Cells were frozen down and stored in liquid nitrogen after 2–5 passages. The schematic illustration of the clonal culture procedure is shown in Supplementary Fig. 1.

#### 5.1.4 Myogenic differentiation

Bulk cultured CD133+ cells were plated onto Matrigel (BD Bioscience, 0.1 mg/ml) coated 8-well chamberslides at a density of 5 × 10^4^ cells/well in M10 medium. Medium was changed to skeletal muscle cell differentiation medium (Promocell) 24 h later to initiate myogenic differentiation. The cultures were fixed 7 days after differentiation. Immunostaining of myosin (MF20, DSHB) was performed and fusion index were evaluated by calculating the percentage of nuclei within the myotubes (defined as myotubes containing at least 3 myonuclei) *versus* total nuclei within the culture.

#### 5.1.5 Intramuscular transplantation of CD133+ cells and analysis of grafted muscles

Two DMD (DMD1 and DMD2) and one normal CD133+ cell preparations (Normal 1, see Table 1) were used to evaluate their contribution to muscle regeneration and to the muscle stem cell pool. On the day of transplantation, 4–8 week-old Rag2-/γ chain-/C5- mice were anesthetized using Isoflurane. Tibialis anterior (TA) muscles were cryodamaged with 3 freeze-thaw cycles using a cryo-probe pre-chilled in liquid nitrogen (Brimah et al., 2004). 5 × 10^5^ hCD133+ cells/5 μl culture medium were injected into each TA with a Hamilton syringe.

Grafted TA muscles were dissected 4 weeks after transplantation and frozen in isopentane chilled in liquid nitrogen. 10 μm transverse cryosections were taken throughout the muscle and stained with antibodies to human spectrin (Vector labs, VP-S283, 1:100, Peterborough, UK), human lamin A/C (Vector labs, VP-L550, 1:100, Peterborough, UK), and Pax7 (DSHB, Iowa, US) followed by corresponding secondary antibodies (Thermal Fisher, Paisley, UK). Images were captured with MetaMorph software using a Leica microscope. The number of human lamin A/C+ nuclei, human spectrin+ fibres, human spectrin+ fibres containing human lamin A/C+ nuclei (as a confirmation that the spectrin+ fibres were of donor origin) were counted in representative transverse sections using MetaMorph software to quantify the number of donor cells and their contribution to muscle regeneration.

The data were analysed by one-way ANOVA or Mann-Whitney test using Graphpad Prism5 software.

#### 5.1.6 RT-PCR analysis

mRNA from cell colonies were extracted using RNAeasy kit (Qiagen) according to manufacturer's instructions. Semi-quantitive RT-PCR was performed using one-step RT-PCR kit (Qiagen) with primers of early stem cell markers nanog, KLF4, Sox2; the satellite cell marker Pax7, pericyte markers ALP, NG2, PDGFRβ, CD146, myoendothelial cell markers CD34, CD56 and CD144. Details of the primers are given in Table 3.

**Table 3:** Primers used for this study.

Name of primer	Sequence-Forward	Sequence-Reverse	Product size
CD133	TCAGTGAGAAAGTGGCATCG	TGTTGTGATGGGCTTGTCAT	313 bp
Nanog	CAATGGTGTGACGCAGAAGG	ACATTAAGGCCTTCCCCAGC	342 bp
KLF4	TTACCAAGAGCTCATGCCACC	GGTGTGCCTTGAGATGGGAA	158 bp
Sox2	TTTGTCGGAGACGGAGAAGC	TAACTGTCCATGCGCTGGTT	237 bp
Pax7	ATTAGCCGCGTGCTCAGAAT	ACTGAACCAGACCTGCACAC	333 bp
ALP	ACCCCAAGTACTGGCGAGA	CTGGCTCGAAGAGACCCAAT	804 bp
NG2	GTCCGACGGGCAACACCAGG	CACTGGCCCTGCTTCCACGG	340 bp
PDGFR-β	AGCTCTACAGCAATGCTCTGCC	GGCTGTCACAGGAGATGGTTG	1047 bp
CD146	CTCCGCGTCTACAAAGCTCC	AGGCCCTGACATTCATAGCG	581 bp
CD34	AGAAAGGCTGGGCGAAGACCCT	AGTGGGGAAGGGTTGGGCGT	311 bp
CD56	GCGTAGCCATGCCCGTGTGT	GCACCTGGGCTGTGCTGGAG	470 bp
CD144	ATCAAGCCCATGAAGCCTCT	TGGTATGCTCCCGGTCAAAC	691 bp
GAPDH	CCCATCACCATCTTCCAGGA	TTGTCATACCAGGAAATGAGC	731 bp

#### 5.1.7 Immunofluorescent staining

Cells were plated at 2 × 10^4^ cells/well onto 8-well chamberslides and fixed 24 h later with 4% paraformaldehyde (PFA) for 15 min. Cells were rinsed with PBS and blocked with PBS containing 10% normal goat serum and 0.03% Triton ×100 for one hour. Then the cells were incubated with primary antibodies against Pax7 (1:100, DSHB), Desmin (1:100, DAKO) and α-SMA (1:100, DAKO) for 2 h at room temperature followed by one hour incubation with Alexa-488- or 594 conjugated isotype matched secondary antibody 1:1000, Thermal Fisher) for one hour. Cells were mounted with mounting medium (DAKO) containing 10 μg/ml DAPI.

The following are the supplementary data related to this article.
